# Can wearables outscore general practitioners? Congenital long QT syndrome diagnosis initiated by a smartwatch

**DOI:** 10.1016/j.hrcr.2024.07.001

**Published:** 2024-07-04

**Authors:** Boris Rudic, Silke Kauferstein, Ibrahim Akin, Martin Borggrefe

**Affiliations:** ∗ I. Department of Internal Medicine, Cardiology, Angiology, Haemostaseology, and Medical Intensive Care, Medical Centre Mannheim, Medical Faculty Mannheim, Heidelberg University, Heidelberg, Germany; † Department of Legal Medicine, University Hospital Frankfurt, Goethe University, Frankfurt am Main, Germany

**Keywords:** Smartwatch, QT measurement, Long QT syndrome, Genetics, *KCNH2*, Withings, ScanWatch

**Key Teaching Points**

• Wearables such as smartwatches can monitor beyond heart rate and heart rhythm.
• Specific smartwatches provide reliable measurements of electrocardiographic intervals (eg, QT interval).
• Correct analysis and interpretation of the QT interval in an individual with previously unknown long QT syndrome facilitated the diagnosis.

## Introduction

### Case report

**Figure 1:**
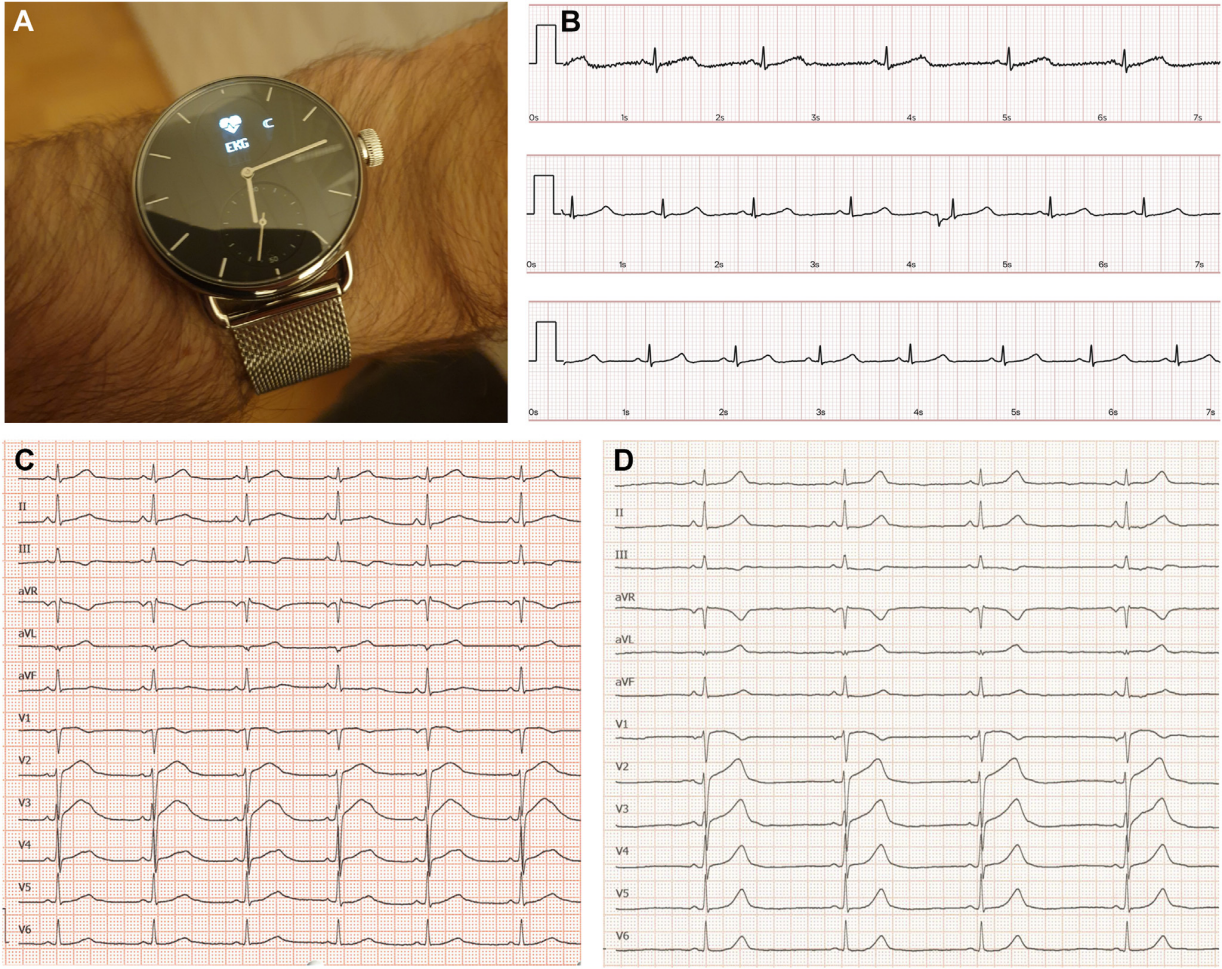
**A:** Withings ScanWatch (Withings SA, Issy les Moulineaux, France) with QT monitoring function; patient is wearing the watch on the left wrist. When the right finger/hand is placed on the bezel, the smartwatch records the lead I electrocardiogram (ECG). **B:** Representative examples of recorded smartwatch ECGs, showing lead DI; 25 mm/s, 10 mm/mV. First panel: QT = 506 ms, heart rate (HR) = 49 beats per minute (bpm), QTc = 457 ms; second panel: QT = 463 ms, HR = 61 bpm, QTc = 466 ms; third panel: QT = 443 ms, HR = 67 bpm; QTc = 468 ms. **C:** A 12-lead ECG with QT prolongation and large prominent T waves in precordial leads; QT: 540 ms, QTc(Bazett): 522 ms, QTc(Framingham): 528 ms, HR: 56 bpm; 25 mm/s, 10 mm/mV. **D:** Normalized QT intervals on beta-blocker therapy with asymptomatic sinus bradycardia; QT: 540 ms, QTc(Bazett): 418 ms, QTc(Framingham): 437 ms, HR: 36 bpm; 25 mm/s, 10 mm/mV.

We report here on a case in which diagnosis of congenital long QT syndrome was facilitated by a smartwatch. A 39-year-old otherwise healthy male patient presented in our outpatient clinic owing to QT prolongation recorded with his smartwatch (ScanWatch; Withings SA, Issy les Moulineaux, France) (Figure 1A and 1B). His medical history was uneventful. He declined palpitations, dyspnea, syncopes, seizures, and family history of sudden cardiac death. He reported to have 2 healthy children aged 7 and 10 years. The smartwatch electrocardiogram (ECG) recordings were acquired out of curiosity and not for specific medical symptoms.

Upon first presentation, the patient showed marked QT prolongation in the resting ECG (QTc 522 ms) and sinus bradycardia (Figure 1C), with a slight underestimation of the QT interval when comparing the smartwatch QT intervals with 12-lead QT intervals. The finding was accentuated at brisk tachycardia during stand-up test with QTc interval >550 ms and notching of the T wave in precordial ECG leads. A “Schwartz score” of 4 was calculated and the electromechanical window assessed by echocardiography showed a strongly negative electromechanical dispersion (-85 ms). Genetic screening for long QT syndrome showed a previously unpublished class 4 nonsense variant in the *KCNH2* gene, resulting in frameshift and protein truncation (NM_000238.3:c.2837delC). Treatment with nadolol 40 mg once a day was prescribed, which resulted in bradycardia during resting phases of 35–40 beats per minute, without reported clinical side effects. Nadolol shortened the QTc interval by 50–70 ms to 430 ms in repeated measurements and reduced the electromechanical window (*Δ* -35 ms). The patient is currently followed at regular intervals without further interventions.

When segregation analysis was performed in family members, the patient’s daughter was identified as a carrier of the same genetic variant, with a borderline prolonged QTc of 460 ms. At the age of 7 she was asymptomatic for syncope, seizures, or bradycardia, and nadolol 1 mg/kg was prescribed after establishing the diagnosis. Her QT interval also shortened during therapy. Screening of the patient’s parents excluded the presence of the *KCNH2* variant in the patient’s father. His mother refused genetic testing.

## Discussion

Long QT syndrome is considered a rare genetic arrhythmia disease and might be overseen in borderline QT prolongation in the range of QTc 440–480 ms and in the absence of pathologic T waves.^1^ Wide distribution and population acceptance of wearables, such as the one presented in this case report, have the potential to identify patients at risk. Several studies have previously validated the use of QT measurement of wearables such as the Apple Watch,^2^ Withings ScanWatch,^3^ and KardiaMobile 6L,^4^ not only in healthy volunteers but also in cardiac patients. These studies generally demonstrated a good level of agreement between smartwatch-calculated QTc interval and the QTc interval obtained from 12-lead ECG. The largest published study, evaluating the accuracy of the automated QTc measurement of a Withings ScanWatch in 367 patients presenting for cardiac work-up, found considerable disagreement (>30 ms) between manually measured QTc and the smartwatch ECG in only 7% of cases. In the remaining 93% of cases, the disagreement between the smartwatch QTc measurement and manually measured QTc was less than 30 ms, demonstrating a sufficient diagnostic accuracy of the Withings ScanWatch.^5^

Secondly, increased availability of next-generation sequencing allows to identify not only causative mutations but also variants of unknown significance.^6^ In our case, a causative nonsense *KCNH2* mutation was accidentally diagnosed in an otherwise heathy patient, only because of prolonged QT intervals measured by his smartwatch. This led to the finding of the same variant in his daughter, diagnosing her with long QT syndrome as well. Females with LQT-2 have higher risk of life-threatening events during postpuberty, pregnancy, and postpartum periods.^7^^,^^8^ Knowledge of the underlying long QT syndrome and avoidance of QT-prolonging drugs, as well as treatment with beta-blockers, could help to reduce burden of adverse cardiac events in this family.

## Disclosures

All authors declare that they have no conflicts of interest.
